# Development and validation of immune inflammation–based index for predicting the clinical outcome in patients with nasopharyngeal carcinoma

**DOI:** 10.1111/jcmm.15097

**Published:** 2020-06-30

**Authors:** Xiaojiao Zeng, Guohong Liu, Yunbao Pan, Yirong Li

**Affiliations:** ^1^ Department of Laboratory Medicine Zhongnan Hospital of Wuhan University Wuhan University Wuhan China; ^2^ Department of Radiology Zhongnan Hospital of Wuhan University Wuhan University Wuhan China

**Keywords:** inflammation indicators, nasopharyngeal carcinoma, neutrophil‐to‐lymphocyte ratio, platelet‐lymphocyte ratio, systemic immune‐inflammation index, systemic inflammation response index

## Abstract

Inflammation indicators, such as systemic inflammation response index (SIRI), systemic immune‐inflammation index (SII), neutrophil‐to‐lymphocyte ratio (NLR) and platelet‐lymphocyte ratio (PLR), are associated with poor prognosis in various solid cancers. In this study, we investigated the predictive value of these inflammation indicators in nasopharyngeal carcinoma (NPC). This retrospective study involved 559 patients with NPC and 500 patients with chronic rhinitis, and 255 NPC patients were followed up successfully. Continuous variables and qualitative variables were measured by t test and chi‐square test, respectively. The optimal cut‐off values of various inflammation indicators were determined by receiver operating characteristic (ROC) curve. Moreover, the diagnostic value for NPC was decided by the area under the curves (AUCs). The Kaplan‐Meier methods and the log‐rank test were used to analyse overall survival (OS) and disease‐free survival (DFS). The independent prognostic risk factors for survival and influencing factors of side effects after treatment were analysed by Cox and logistic regression analysis, respectively. Most haematological indexes of NPC and rhinitis were significantly different between the two groups, and PLR was optimal predictive indicators of diagnosis. In the multivariable Cox regression analysis, PLR, WBC, RDW, M stage and age were independent prognostic risk factors. Many inflammation indicators that affected various side effects were evaluated by logistic regression analysis. In conclusion, the combined inflammation indicators were superior to single haematological indicator in the diagnosis and prognosis of NPC. These inflammation indicators can be used to supply the current evaluation system of the TNM staging system to help predict the prognosis in NPC patients.

## 1 INTRODUCTION

Nasopharyngeal carcinoma (NPC) is a malignant epithelial cancer that occurs in the epithelial lining of the nasopharynx with the highest rate of metastasis among head and neck cancers.^1^ NPC has an extraordinarily skewed geographic distribution worldwide, which is mainly prevalent in southern China and South‐East Asian countries.^1^ More than 129 000 new cases of NPC were reported worldwide, and the incidence of the male is higher than that of female.^1^ The mortality from cancer is mostly attributable to metastases, not the primary cancers, and the effective treatment for cancer depends mainly on our capacity to reverse the process of metastasis.^2^ Intensity‐modulated radiation therapy (IMRT) and concurrent chemotherapy are regarded as the primary treatment for NPC.^3^ However, the treatment is related to acute and late toxicities with impairment of patients’ quality of life,^4^ such as dysphagia.^5^, ^6^ Other side effects, such as the arrest of bone marrow, radiation stomatitis and dermatitis, need to be further investigated.

The classification method of NPC is mainly relied on the tumour‐node‐metastasis (TNM) staging criteria, which is used for treatment selection, cancer control activities and outcome prediction. However, the failure to consider the functional status of NPC leads to different prognoses in patients with the same TNM staging.^7^ More reliable markers are necessary to supply clinical diagnosis and treatment.

The inflammatory responses play an essential role in various stages of cancer development, including occurrence, progression, malignant conversion, invasion and metastasis, and moreover, the inflammation affects immune surveillance and responses to therapy.^8^ Solid malignancies trigger an intrinsic inflammatory response and then building up a pro‐tumorigenic microenvironment, which promotes the development of cancers.^9^ Cancers contain various noncancerous cells including immune cells, such as T cells, macrophages and neutrophils. These cells can be anti‐ or tumorigenic and associate with survival in several cancer types.^10^

The inflammation indicators including neutrophils,^11^ lymphocytes and monocytes,^12^ and red cell volume distribution width (RDW)^13^ have prognostic value in cancers. The integration of two types of white blood cell indicators, such as the neutrophil‐lymphocyte ratio (NLR), platelet‐lymphocyte ratio (PLR) and lymphocyte‐monocyte ratio (LMR), is considered to be independent prognostic factors for colorectal cancer.^14^ Recently, immune‐inflammation indexes including the systemic inflammatory response index (SIRI) based on three types of white cells (peripheral neutrophils, monocytes and lymphocytes) and the systemic immune‐inflammation index (SII) based on three types of white cells (peripheral neutrophils, platelet and lymphocytes) were investigated in various cancers.^15^, ^16^ These inflammation indexes are also considered to be independent prognostic factors for cancers, and their prognostic value is higher than that of only white blood cells. However, the cut‐off value of immune‐inflammation indicators is diverse in different cancers. The cut‐off value of SII, NLR and PLR in non–small‐cell lung cancer is 660, 3.57 and 147, respectively,^16^ while these values in metastatic prostate cancer are 535, 3 and 210, respectively.^17^ There are few reports on the relationship between combined inflammation indicators and NPC prognosis, and the basophil has never been reported in NPC prognosis.

In this study, we investigated the efficiency of these inflammation indicators on the diagnosis of NPC, and these inflammation indicators can be established as a mechanism for predicting prognosis of cancer patients in clinical settings that would help for future novel treatments.

## 2 MATERIALS AND METHODS

### 2.1 Patients

We retrospectively recruited 559 patients who were diagnosed as NPC at the Zhongnan Hospital of Wuhan University from January 2014 to November 2018. NPC patients were comprised by 421 males and 138 females with a median age of 51 (range 12‐83 years). To verify the predictive value of the immune‐inflammation indicators for diagnosis of NPC, we retrospectively recruited other 500 patients diagnosed as rhinitis in the same period as normal group who were comprised by 312 males and 188 females with a median age of 46 (range 10‐83 years). The seventh edition of the American Joint Committee on Cancer (AJCC) staging system was used for stage classification. This study was carried out in accordance with the recommendations of Zhongnan Hospital of Wuhan University Ethics and Scientific Committee with written informed consent from all patients. All patients gave written informed consent in accordance with the Declaration of Helsinki. The protocol was approved by the Zhongnan Hospital of Wuhan University Ethics and Scientific Committee.

### 2.2 Inclusion and exclusion criteria

The inclusion criteria in this study comprised of: (a) patients with histopathological confirmation of NPC; (b) patients with proper renal, cardiac and liver function to tolerate chemotherapy and radiotherapy; and (c) patients with a complete record of haematological indicators. Exclusion criteria were as follows: (a) patients with other types of malignancy. Finally, we have retrieved data of 255 patients with complete follow‐up data using for survival analysis.

### 2.3 Haematological examination

Fasting whole blood from every patient was collected in an EDTA anticoagulant‐treated tube on the admission without the next step of treatment, and analysed within 30 minutes of collection. Routine peripheral blood cells, including total white cell count (WBC), red blood cell count (RBC), platelet count (PLT), differential white cell count (neutrophils, lymphocytes, monocytes, eosinophils and basophils), haemoglobin (HGB), haematocrit (HCT), mean cell volume (MCV), mean cell haemoglobin (MCH), mean cell haemoglobin concentration (MCHC), red cell distribution width (RDW) and mean platelet volume (MPV), were analysed by Beckman Coulter DxH 800 automated blood analyser and related reagents (Beckman, California, USA). The combination of two or three haematological inflammation parameters, SIRI, SII, NLR and PLR, is defined as follows:

SIRI = neutrophils × monocytes/lymphocytes;
NLR = neutrophils/lymphocytes;
SII = neutrophils × platelets/lymphocytes;
PLR = platelets/lymphocytes;

ROC curves determined the optimal cut‐off values for prognostic inflammation indicators (area under the curve > 50%).^18^ The optimal cut‐off values were as follows: SIRI (1.529), NLR (3.441), SII (715.739), PLR (245.496), neutrophil (2.722), monocyte (0.578), platelet (267.583), WBC (6.177), basophil (0.029) and RDW (14.495).

### 2.4 Follow‐up

We chose the OS and DFS as the primary end‐point and secondary end‐point, respectively. Patients diagnosed as NPC were followed up primarily by telephone and periodic review in hospital. A total of 255 of 559 patients were followed up successfully. OS was defined as the period from the initial diagnosis to death regardless of NPC related or not or the last follow‐up. The median follow‐up time among the 255 patients was 33.5 months, ranging from 2.1 months to 151.2 months. DFS was defined as the period from the initial diagnosis to recurrence or metastasis. Follow‐ups were ended in February 2019.

### 2.5 Statistical analysis

Statistical analyses were conducted using IBM SPSS version 22.0 software (SPSS, Chicago, IL). Continuous variables and qualitative variables were measured by t test and chi‐square test and plotted by GraphPad Prism V7.0 software. The correlations between clinical factors and SIRI, SII, NLR, neutrophil and monocyte were analysed by chi‐square test. The Kaplan‐Meier methods and the log‐rank test were used to estimate OS and DFS. The independent prognostic risk factors for survival were analysed by univariate and multivariate Cox proportional hazards regression model. The logistic regression analysis was used to analyse the influencing factors of side effects after treatment. Receiver operating characteristic (ROC) curve was applied to determine optimal cut‐off values and assess the predictive ability of prognostic indicators.^19^ A *P*‐value < .05 was considered statistically significant.

## 3 RESULTS

### 3.1 Baseline characteristics of NPC and rhinitis patients

**Figure 1:**
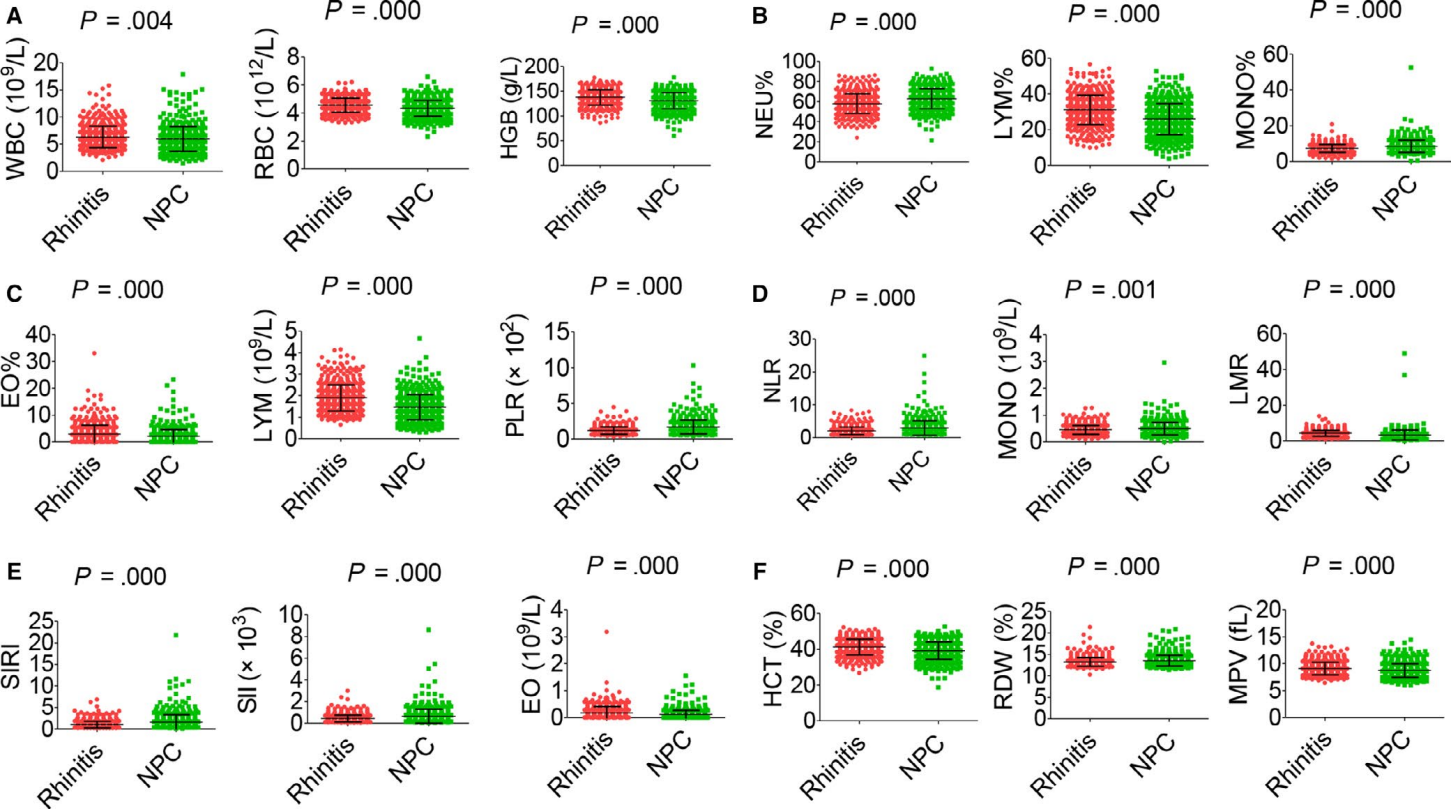
General characteristics of haematological parameters between NPC and rhinitis patients. A, WBC (left), RBC (middle) and HGB (right). B, NEU% (left), LYM% (middle) and MONO% (right). C, EO% (left), LYM (middle) and PLR (right). D, NLR (left), MONO (middle) and LMR (right). E, SIRI (left), SII (middle) and EO (right). F, HCT (left), RDW (middle) and MPV (right)

**Figure 2:**
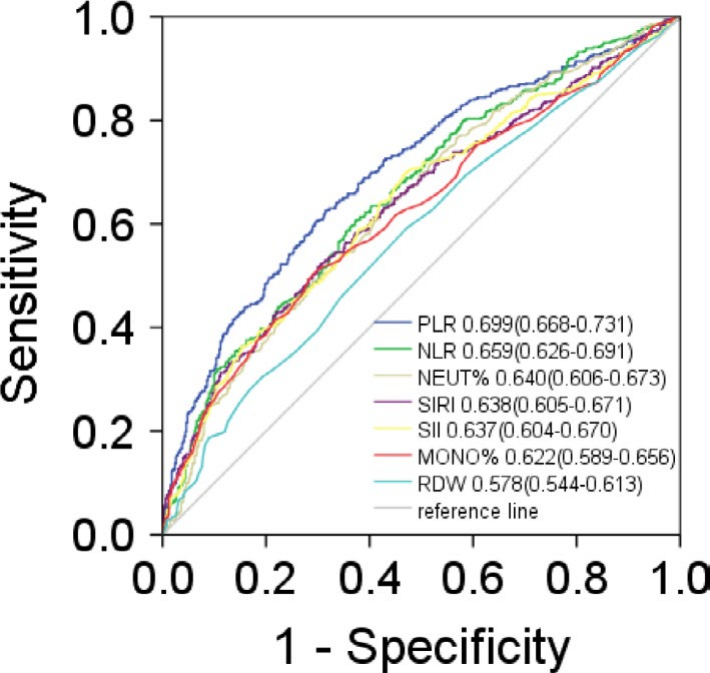
The diagnostic significance of immunological indexes was analysed via establishing ROC curve in NPC. The curve demonstrated that immunological indexes could discriminate NPC from rhinitis

NPC and rhinitis were both common in men and younger patients (Table 1). Clinical parameters between NPC patients and rhinitis patients are shown in Figure 1. Most immune‐inflammation indicators between two cohorts, such as PLR, NLR, SIRI and SII, were significantly different. To investigate the diagnostic significance of immunological indexes in NPC patients, ROC curve analysis was performed. As shown in Figure 2, the AUC values for PLR, NLR, NEU, SIRI, SII and MONO were 0.699, 0.659, 0.640, 0.638, 0.637 and 0.622, while the AUC value for RDW was 0.578. These data suggested that PLR NLR, SIRI, SII, NEU and MONO could distinguish NPC from rhinitis.

**Table 1:** General characteristics of NPC and rhinitis cohort

Variables	All patients		NPC with follow‐up
	NPC, n = 559	Rhinitis, n = 500	n = 255
Sex			
Male	421 (75.3%)	312 (62.4%)	202 (79.2%)
Female	138 (24.7%)	188 (37.6)	53 (20.8%)
Age			
<60	422 (75.5%)	410 (82.0%)	193 (75.7%)
≥60	137 (24.5%)	90 (18.0%)	62 (24.3%)
T			
T1	65 (11.6%)	n.a.	33 (12.9%)
T2	166 (29.7%)	n.a.	70 (27.5%)
T3	162 (29%)	n.a.	70 (27.5%)
T4	166 (29.7%)	n.a.	82 (32.1%)
N			
N0	43 (7.7%)	n.a.	18 (7.1%)
N1	91 (16.3%)	n.a.	43 (16.8%)
N2	338 (60.5%)	n.a.	156 (61.2%)
N3	87 (15.5%)	n.a.	38 (14.9%)
M			
M0	492 (88%)	n.a.	231 (90.6%)
M1	67 (12%)	n.a.	24 (9.4%)
Histology (WHO)			
Keratinizing	12 (2.1%)	n.a.	6 (2.4%)
Non‐keratinizing	527 (94.3%)	n.a.	243 (95.2%)
Unknown	20 (3.6%)	n.a.	6 (2.4%)

Note

Abbreviations: TNM, tumour node metastasis; n.a, not applicable; WHO, World Health Organization.

Keratinizing squamous cell carcinoma; Non‐keratinizing carcinoma.

### 3.2 The association between clinical indexes and haematological indicators in NPC patients

**Figure 3:**
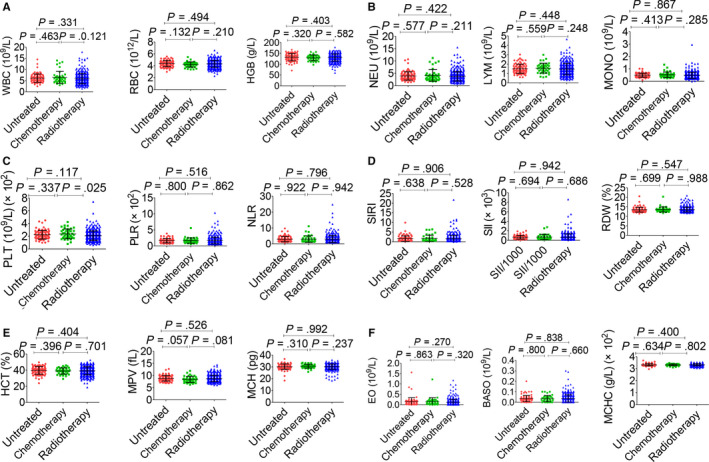
Effects of therapy on haematological parameters. A, WBC (left), RBC (middle) and HGB (right). B, NEU (left), LYM (middle) and MONO (right). C, PLT (left), PLR (middle) and NLR (right). D, SIRI (left), SII (middle) and RDW (right). E, HCT (left), MPV (middle) and MCH (right). F, EO (left), BASO (middle) and MCHC (right). Radiotherapy included the chemoradiotherapy and radiotherapy alone

**Figure 4:**
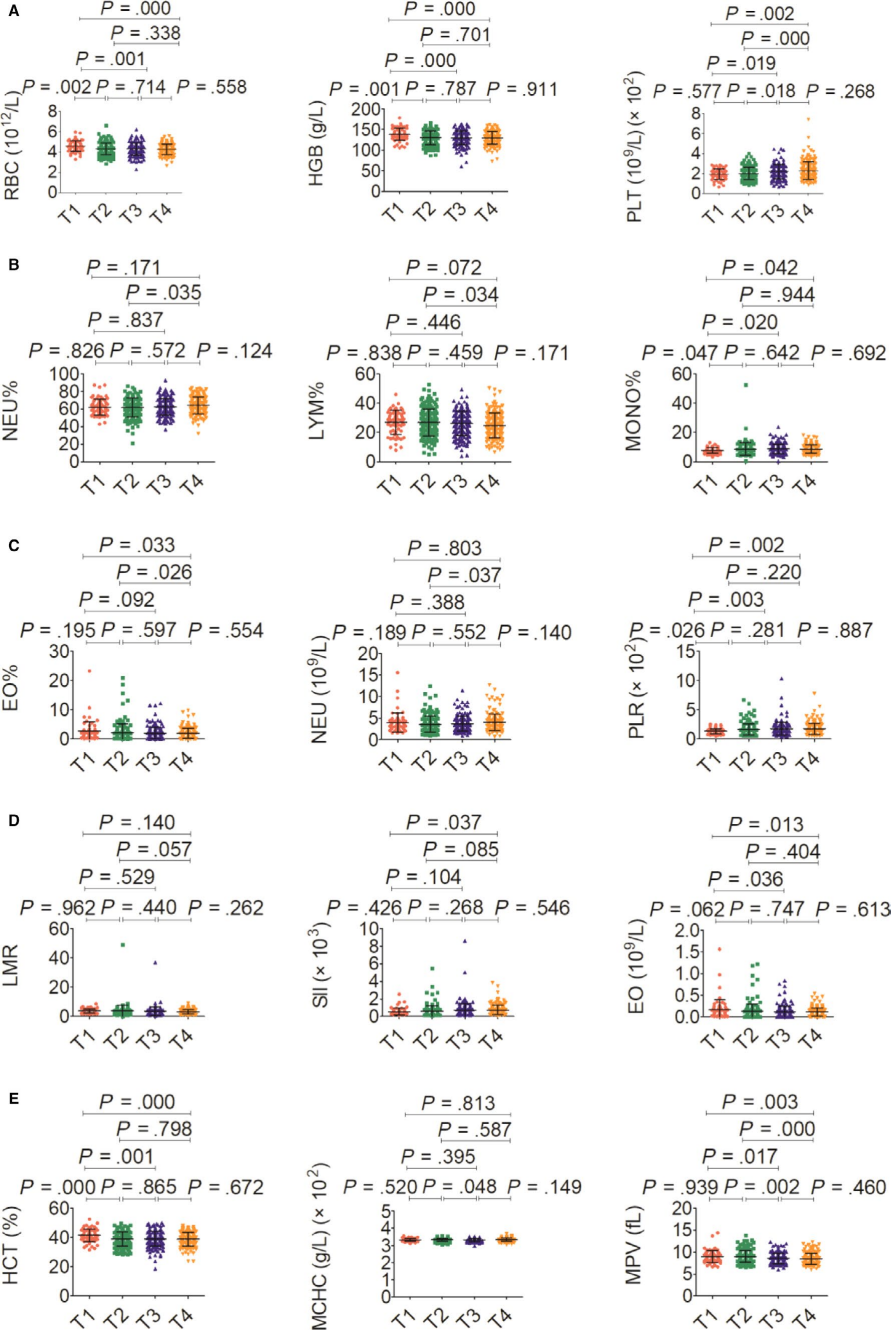
Effects of T stage on haematological parameter. A, RBC (left), HGB (middle) and PLT (right). B, NEU% (left), LYM% (middle) and MONO% (right). C, EO% (left), NEU (middle) and PLR (right). D, LMR (left), SII (middle) and EO (right). E, HCT (left), MCHC (middle) and MPV (right)

**Figure 5:**
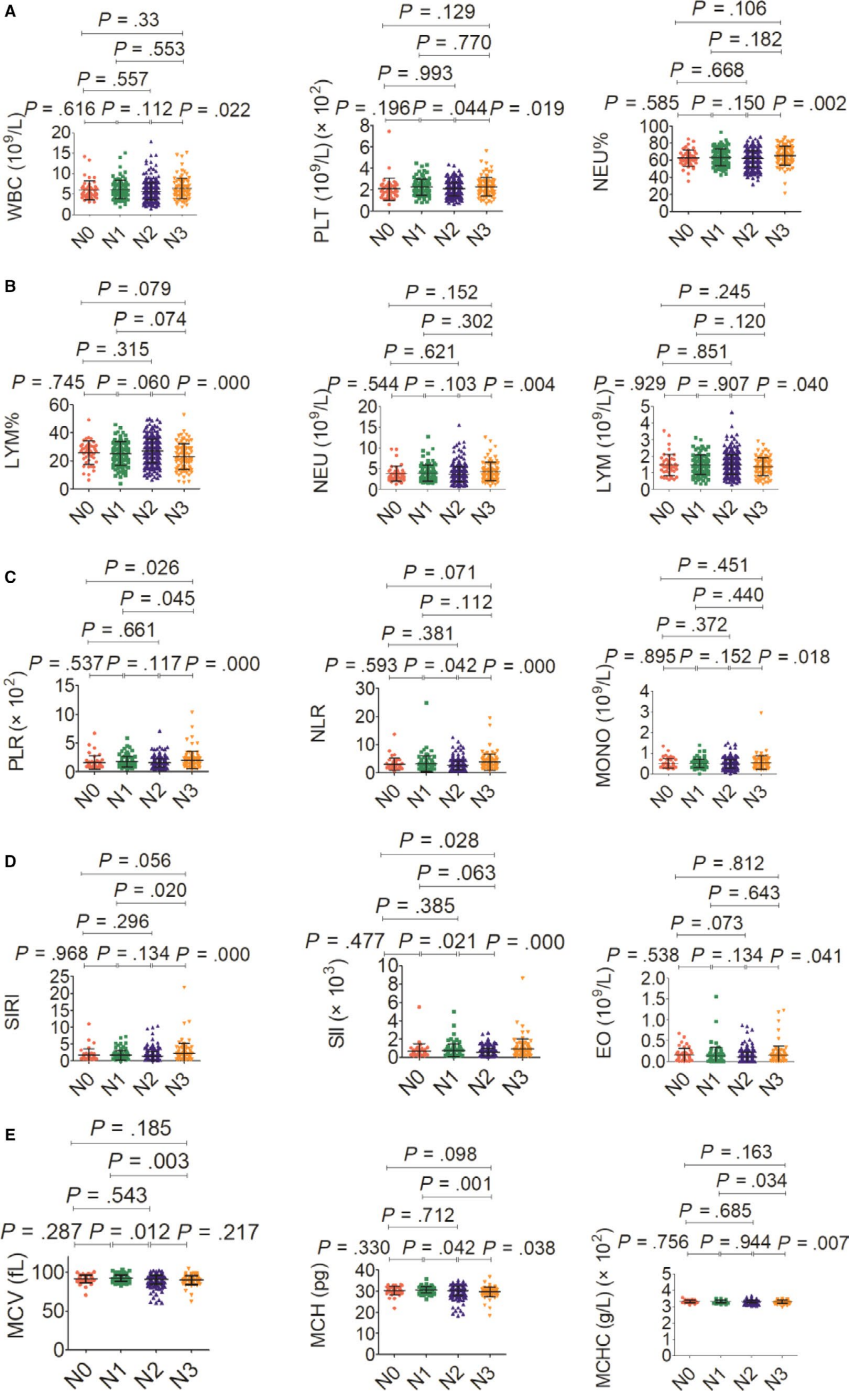
Effects of N stage on haematological parameter. A, WBC (left), PLT (middle) and NEU% (right). B, LYM% (left), NEU (middle) and LYM (right). C, PLR (left), NLR (middle) and MONO (right). D, SIRI (left), SII (middle) and EO (right). E, MCV (left), MCH (middle) and MCHC (right)

**Figure 6:**
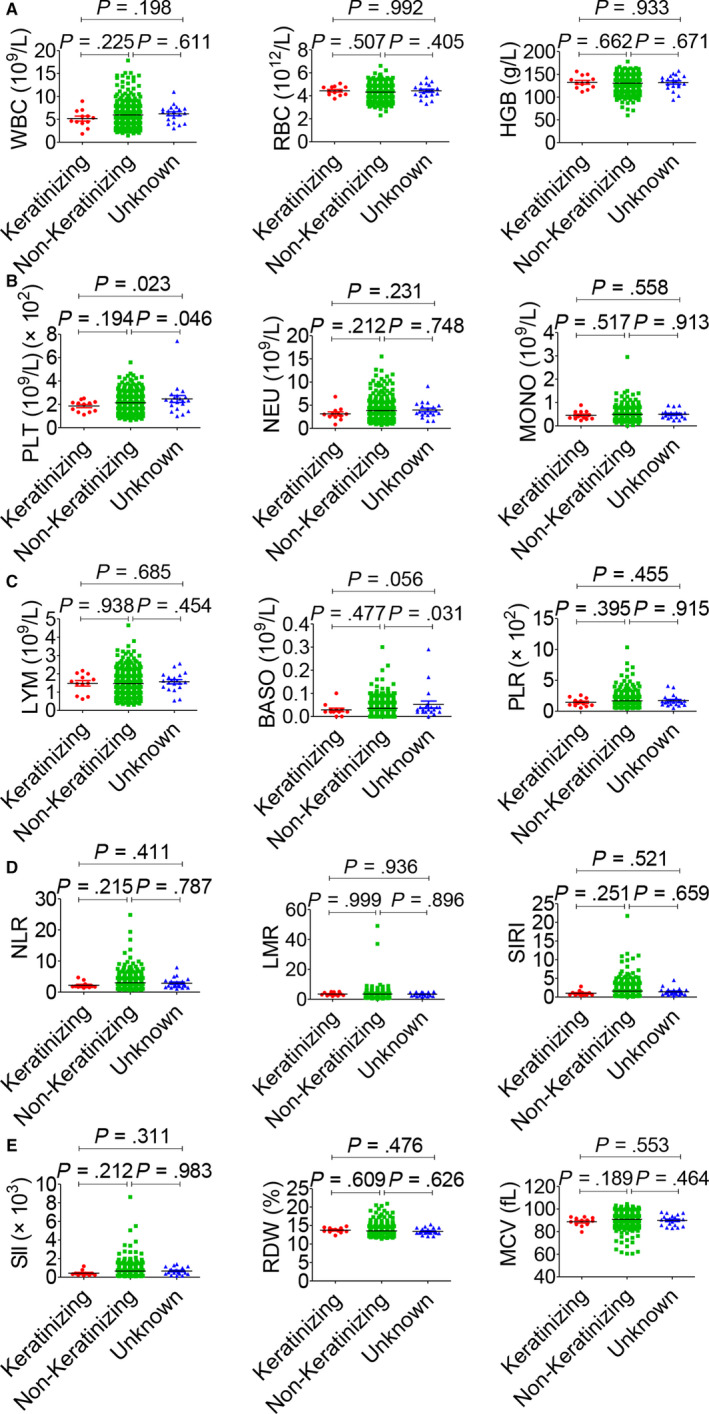
Effects of pathological type on haematological parameters. A, WBC (left), RBC (middle) and HGB (right). B, PLT (left), NEU (middle) and MONO (right). C, LYM (left), BASO (middle) and PLR (right). D, NLR (left), LMR (middle) and SIRI (right). E, SII (left), RDW (middle) and MCV (right)

The association between haematological indicators and clinical characteristics in 559 NPC patients was shown in Table 2, and haematological indicators in a different circumstance, including therapy, TNM staging system and histopathological classification, were displayed in Figures 3, 4, 5, 6. Significant differences in the haematological indicators were diverse in sex, age and metastasis status (Table 2). Moreover, common differences in inflammation indicators (such as SII and PLR) in multiple comparative analysis can be observed (Figures 4, 5). However, there were not significant differences in inflammation indicators in therapy and histopathological groups despite the difference in platelets in these groups (Figures 3 and 6).

**Table 2:** General characteristics of haematological parameters of 559 included patients

Parameters	Sex	$\bar{x}\pm s$	*P*	Age	$\bar{x}\pm s$	*P*	M	$\bar{x}\pm s$	*P*
WBC	M	6.156 ± 2.313	.000	<60	5.958 ± 2.249	0.828	M0	5.923 ± 2.295	.570
	F	5.307 ± 1.898		≥60	5.910 ± 2.245		M1	6.116 ± 2.600	
RBC	M	4.433 ± 0.557	.000	<60	4.385 ± 0.579	.000	M0	4.363 ± 0.559	0.002
	F	4.040 ± 0.471		≥60	4.187 ± 0.480		M1	4.139 ± 0.554	
HGB	M	134.065 ± 15.321	.000	<60	131.281 ± 16.645	0.116	M0	131.745 ± 15.585	.000
	F	120.291 ± 14.562		≥60	128.766 ± 14.867		M1	122.736 ± 18.793	
PLT	M	211.572 ± 72.830	0.264	<60	219.929 ± 73.449	.000	M0	210.878 ± 70.164	0.051
	F	219.565 ± 72.707		≥60	193.883 ± 67.371		M1	233.134 ± 88.097	
NEU%	M	63.033 ± 9.828	0.096	<60	62.516 ± 10.038	0.621	M0	62.098 ± 9.743	0.001
	F	61.415 ± 10.132		≥60	62.999 ± 9.572		M1	66.570 ± 10.383	
LYM%	M	25.502 ± 8.647	0.016	<60	26.342 ± 8.670	0.114	M0	26.632 ± 8.569	.000
	F	27.561 ± 8.703		≥60	24.989 ± 8.737		M1	21.443 ± 8.324	
MONO%	M	8.713 ± 2.900	0.139	<60	8.441 ± 3.539	0.063	M0	8.538 ± 3.425	0.309
	F	8.222 ± 4.541		≥60	9.058 ± 2.804		M1	8.987 ± 3.037	
EO%	M	2.106 ± 2.169	.750	<60	2.070 ± 2.408	0.351	M0	2.111 ± 2.311	0.781
	F	2.182 ± 3.081		≥60	2.293 ± 2.472		M1	2.222 ± 3.145	
BASO%	M	0.648 ± 0.746	0.678	<60	0.633 ± 0.745	0.653	M0	0.623 ± 0.436	.460
	F	0.620 ± 0.453		≥60	0.664 ± 0.457		M1	0.769 ± 1.595	
NEUT	M	3.979 ± 1.973	0.001	<60	3.822 ± 1.921	0.992	M0	3.770 ± 1.862	0.124
	F	3.344 ± 1.594		≥60	3.820 ± 1.863		M1	4.203 ± 2.171	
LYM	M	1.496 ± 0.596	0.081	<60	1.496 ± 0.593	0.079	M0	1.504 ± 0.571	.000
	F	1.397 ± 0.528		≥60	1.396 ± 0.537		M1	1.234 ± 0.605	
PLR	M	163.952 ± 99.331	0.118	<60	170.324 ± 95.494	.250	M0	158.390 ± 78.420	.000
	F	178.850 ± 89.078		≥60	159.333 ± 101.549		M1	235.486 ± 168.960	
NLR	M	3.079 ± 2.291	0.051	<60	2.926 ± 2.144	0.331	M0	2.811 ± 1.775	0.004
	F	2.667 ± 1.644		≥60	3.132 ± 2.191		M1	4.197 ± 3.758	
MONO	M	0.518 ± 0.215	.000	<60	0.484 ± 0.237	0.106	M0	0.490 ± 0.231	.330
	F	0.418 ± 0.261		≥60	0.521 ± 0.209		M1	0.519 ± 0.233	
LMR	M	3.264 ± 2.171	0.005	<60	3.510 ± 2.613	0.395	M0	3.483 ± 2.084	0.483
	F	4.029 ± 4.112		≥60	3.276 ± 3.295		M1	3.228 ± 5.799	
SIRI	M	1.687 ± 1.821	0.001	<60	1.488 ± 1.534	0.119	M0	1.456 ± 1.449	0.016
	F	1.197 ± 1.364		≥60	1.807 ± 2.220		M1	2.375 ± 2.992	
SII	M	676.431 ± 695.005	0.228	<60	663.442 ± 592.547	0.709	M0	604.570 ± 486.474	0.005
	F	600.356 ± 449.140		≥60	639.811 ± 782.321		M1	1047.435 ± 1251.348	
EO	M	0.124 ± 0.147	0.496	<60	0.118 ± 0.149	0.262	M0	0.123 ± 0.152	0.773
	F	0.114 ± 0.176		≥60	0.135 ± 0.170		M1	0.117 ± 0.174	
BASO	M	0.037 ± 0.035	0.165	<60	0.035 ± 0.035	0.694	M0	0.036 ± 0.033	0.934
	F	0.032 ± 0.028		≥60	0.037 ± 0.031		M1	0.036 ± 0.042	
HCT	M	40.298 ± 4.607	.000	<60	39.485 ± 5.019	0.083	M0	39.572 ± 4.706	.000
	F	36.181 ± 4.302		≥60	38.656 ± 4.316		M1	37.155 ± 5.496	
MCV	M	91.171 ± 5.353	0.006	<60	90.266 ± 5.557	.000	M0	90.950 ± 5.314	0.094
	F	89.697 ± 5.628		≥60	92.474 ± 4.772		M1	89.760 ± 6.340	
MCH	M	30.353 ± 2.121	0.013	<60	30.037 ± 2.190	.000	M0	30.302 ± 2.071	0.022
	F	29.833 ± 2.183		≥60	30.803 ± 1.898		M1	29.661 ± 2.579	
MCHC	M	332.794 ± 9.186	.660	<60	332.592 ± 8.963	0.609	M0	333.052 ± 8.661	0.012
	F	332.425 ± 8.311		≥60	333.044 ± 9.024		M1	330.137 ± 10.720	
RDW	M	13.481 ± 1.115	0.062	<60	13.520 ± 1.295	0.371	M0	13.465 ± 1.084	0.007
	F	13.748 ± 1.545		≥60	13.629 ± 1.048		M1	14.148 ± 1.956	
MPV	M	8.627 ± 1.199	0.007	<60	8.711 ± 1.286	0.821	M0	8.747 ± 1.285	0.147
	F	8.996 ± 1.421		≥60	8.739 ± 1.208		M1	8.507 ± 1.105	

Abbreviations: HGB, haemoglobin; PLT, platelet; NEU, neutrophil; LYM, lymphocyte; MONO, monocyte; EO, eosinophil; BASO, basophil; PLR, platelet‐lymphocyte ratio; NLR, neutrophil‐lymphocyte ratio; LMR, lymphocyte‐monocyte ratio; SIRI, systemic inflammation response index; SII, systemic immune‐inflammation index; HCT, haematocrit; MCV, erythrocyte mean corpuscular volume; MCH, erythrocyte mean corpuscular haemoglobin; MCHC, erythrocyte mean corpuscular haemoglobin concentrate; RDW, erythrocyte haemoglobin distribution width; MPV, mean platelet volume.

### 3.3 Influence of clinical indexes and haemograms on side effects

A total of 509 of 559 NPC patients received radiotherapy, but 2 patients of them were deficient in clinical data and therefore excluded in our study. Then, 507 patients were included in the study for side effects (Table S1). Common side effects of treatment in our study consisted of the arrest of bone marrow, radiodermatitis, radiation stomatitis, skin pigmentation after radiotherapy, dysphagia, gastrointestinal reaction and innutrition. Part of these patients was confronted with these side effects, including bacterial infection, secondary anaemia, hypoproteinaemia, post‐radiotherapy moult, electrolyte disturbances, secondary thrombocytopenia, abnormal liver function and agranulocytosis. We conducted a study on the factors affecting the side effects of treatment. Results analysed by multivariate logistic regression analysis are shown in Tables 3, 4, 5, 6. The independent risk factors for the arrest of bone marrow included, lymphocyte, eosinophil, HCT and MCV (Table 3). The independent risk factors for the radiodermatitis included lymphocyte and eosinophil (Table 3), and the independent risk factors for the radiation stomatitis included haemoglobin, platelet, lymphocyte, monocyte, eosinophil and basophil (Table 4). And the independent risk factors for the skin pigmentation after radiotherapy included age, PLR, eosinophil and HCT (Table 4). The independent risk factors for the dysphagia included eosinophil, HCT and PLR (Table 5), and the independent risk factors for the gastrointestinal reaction included sex, SIRI, M stage, eosinophil and HCT (Table 5). Haemoglobin, NLR and age were the independent risk factors for the innutrition (Table 6). Age, eosinophil and HCT affected most side effects in the treatment of NPC patients, while T stage, N stage, histology, neutrophil and SII had no impact on these side effects.

**Table 3:** Effects of clinical parameters and hemograms on the arrest of bone marrow and radiodermatitis in NPC patients (n = 507)

Variables	n	Arrest of bone marrow			Radiodermatitis		
		OR	95% CI	*P*	OR	95% CI	*P*
Sex		1.428	0.845‐2.412	.183	1.234	0.767‐1.986	.385
Male	386	Ref.			Ref.		
Female	121						
Age		1.289	0.777‐2.138	.325	0.669	0.433‐1.034	.070
<60	389	Ref.			Ref.		
≥60	118						
T				.387			.735
T1	62	Ref.			Ref.		
T2	149	0.828	0.400‐1.720	.613	1.220	0.629‐2.366	.556
T3	151	1.227	0.590‐2.549	.584	1.218	0.619‐2.395	.568
T4	145	0.785	0.375‐1.644	.521	0.962	0.489‐1.892	.910
N				.100			.950
N0	40	Ref.			Ref.		
N1	84	0.434	0.183‐1.030	.058	0.817	0.357‐1.866	.631
N2	305	0.494	0.231‐1.059	.070	0.915	0.436‐1.923	.815
N3	78	0.317	0.126‐0.797	.015	0.834	0.351‐1.984	.681
M		1.277	0.670‐2.432	.457	1.635	0.927‐2.885	.090
M0	436	Ref.			Ref.		
M1	71						
Histology				.142			.495
Keratinizing*	12	Ref.			Ref.		
Non‐Keratinizing#	479	0.361	0.099‐1.319	.123	0.414	0.097‐1.777	.235
Unknown	16	0.763	0.141‐4.111	.753	0.417	0.070‐2.492	.337
SIRI		1.219	0.615‐2.414	.571	0.852	0.452‐1.606	.621
<1.529	367	Ref.			Ref.		
≥1.529	140						
NLR		0.922	0.411‐2.068	.844	1.179	0.553‐2.511	.670
<3.441	377	Ref.			Ref.		
≥3.441	130						
SII		0.935	0.432‐2.025	.865	1.069	0.505‐2.263	.861
<715.739	384	Ref.			Ref.		
≥715.739	123						
PLR		1.776	0.903‐3.492	.096	1.158	0.518‐2.589	.720
<245.496	442	Ref.			Ref.		
≥245.496	65						
WBC				.049			.850
Normal	341	Ref.			Ref.		
Low	146	0.531	0.319‐0.885	.015	1.229	0.593‐2.548	.579
High	20	1.038	0.366‐2.945	.944	0.853	0.132‐5.489	.867
RBC				.390			.335
Normal	324	Ref.			Ref.		
Low	178	1.223	0.621‐2.408	.560	1.510	0.832‐2.740	.176
High	5	5.164	0.399‐66.905	.209	0.464	0.044‐4.942	.525
HGB		0.618	0.319‐1.198	.154	0.687	0.382‐1.236	.210
Normal	330	Ref.			Ref.		
Low	177						
PLT				.476			.370
Normal	455	Ref.			Ref.		
Low	15	1.008	0.225‐4.520	.992	1.501	0.420‐5.362	.532
High	37	1.690	0.727‐3.932	.223	1.787	0.735‐4.346	.200
NEU				.591			.268
Normal	370	Ref.			Ref.		
Low	109	0.834	0.380‐1.832	.652	0.557	0.270‐1.148	.112
High	28	0.468	0.091‐2.414	.364	0.769	0.145‐4.089	.758
LYM				.001			.022
Normal	379	Ref.			Ref.		
Low	127	2.939	1.655‐5.218	.000	1.878	1.202‐2.936	.006
High	1	7.951E+09	0‐	.999	0.000	0‐	.999
MONO		1.561	0.682‐3.577	0.292	1.063	0.502‐2.248	.873
Normal	463	Ref.			Ref.		
High	44						
EO				.000			.002
Normal	210	Ref.			Ref.		
Low	290	0.343	0.227‐0.519	.000	0.502	0.342‐0.736	.000
High	7	0.297	0.049‐1.787	.185	1.122	0.206‐6.107	.894
BASO		0.646	0.156‐2.668	.546	0.252	0.062‐1.021	.053
Normal	497	Ref.			Ref.		
High	10						
HCT		0.489	0.317‐0.754	.001	0.861	0.524‐1.412	.553
Normal	154	Ref.			Ref.		
Low	353						
MCV				.002			.954
Normal	483	Ref.			Ref.		
Low	13	6.694	2.002‐22.377	.002	0.922	0.055‐15.414	.955
High	11	3.154	0.831‐11.974	.091	1.277	0.262‐6.217	.762
MCH				.487			.350
Normal	485	Ref.			Ref.		
Low	12	0.325	0.020‐5.256	.428	4.112	0.173‐97.498	.381
High	10	2.246	0.390‐12.924	.365	2.934	0.466‐18.493	.252
MCHC				.545			.619
Normal	481	Ref.			Ref.		
Low	23	1.754	0.565‐5.441	.331	1.744	0.573‐5.309	.327
High	3	0.454	0.024‐8.660	.600	0.971	0.059‐15.998	.984
RDW		0.974	0.475‐1.997	.943	0.849	0.456‐1.581	.606
Normal	436	Ref.			Ref.		
High	71						
MPV		1.190	0.024‐59.676	.931	0.484	0.018‐12.830	.665
Normal	505	Ref.			Ref.		
High	2		‐				

Note

Keratinizing squamous cell carcinoma; non‐keratinizing carcinoma.

**Table 4:** Effects of clinical parameters and hemograms on the radiation stomatitis and skin pigmentation after radiotherapy in NPC patients (n = 507)

Variables	n	Radiation stomatitis			Skin pigmentation after radiotherapy		
		OR	95% CI	*P*	OR	95% CI	*P*
Sex		1.211	0.740‐1.984	.446	0.942	0.576‐1.540	.811
Male	386	Ref.			Ref.		
Female	121						
Age		0.656	0.416‐1.036	.070	1.656	1.027‐2.671	.039
<60	389	Ref.			Ref.		
≥60	118						
T				.258			.348
T1	62	Ref.			Ref.		
T2	149	1.666	0.845‐3.285	.141	0.728	0.362‐1.464	.373
T3	151	1.869	0.931‐3.750	.078	1.176	0.573‐2.415	.658
T4	145	1.306	0.652‐2.613	.451	0.910	0.444‐1.865	.797
N				.645			.454
N0	40	Ref.			Ref.		
N1	84	0.804	0.343‐1.885	.616	0.846	0.332‐2.157	.726
N2	305	0.966	0.448‐2.082	.929	0.643	0.277‐1.491	.303
N3	78	0.676	0.275‐1.664	.394	0.936	0.354‐2.476	.894
M		1.503	0.789‐2.862	.215	1.263	0.654‐2.439	.487
M0	436	Ref.			Ref.		
M1	71						
Histology				.389			.699
Keratinizing*	12	Ref.			Ref.		
Non‐Keratinizing#	479	0.913	0.233‐3.585	.897	1.398	0.394‐4.967	.604
Unknown	16	2.192	0.354‐13.578	.399	0.948	0.181‐4.947	.949
SIRI		0.545	0.294‐1.010	.054	1.213	0.618‐2.383	.574
<1.529	367	Ref.			Ref.		
≥1.529	140						
NLR		1.798	0.904‐3.578	.095	1.200	0.533‐2.705	.659
<3.441	377	Ref.			Ref.		
≥3.441	130						
SII		0.785	0.356‐1.730	.548	0.862	0.385‐1.929	.718
<715.739	384	Ref.			Ref.		
≥715.739	123						
PLR		1.341	0.570‐3.158	.501	3.379	1.696‐6.731	.001
<245.496	442	Ref.			Ref.		
≥245.496	65						
WBC				.229			.082
Normal	341	Ref.			Ref.		
Low	146	0.603	0.286‐1.268	.182	0.743	0.349‐1.584	.442
High	20	3.175	0.468‐21.537	.237	8.051	1.209‐53.639	.031
RBC				.201			.282
Normal	324	Ref.			Ref.		
Low	178	1.597	0.860‐2.964	0.138	1.667	0.888‐3.126	.112
High	5	0.277	0.027‐2.884	0.283	1.026E+09	0‐	.999
HGB		0.537	0.357‐0.809	0.003	0.646	0.348‐1.198	.165
Normal	330	Ref.			Ref.		
Low	177						
PLT				.008			.430
Normal	455	Ref.			Ref.		
Low	15	1.258	0.411‐3.849	.688	2.534	0.620‐10.355	.195
High	37	4.547	1.743‐11.861	.002	1.074	0.441‐2.618	.875
NEU				.080			.067
Normal	370	Ref.			Ref.		
Low	109	0.680	0.417‐1.110	.123	1.594	0.746‐3.405	.229
High	28	0.456	0.175‐1.185	.107	0.179	0.034‐0.945	.043
LYM				.008			.281
Normal	379	Ref.			Ref.		
Low	127	2.325	1.365‐3.960	.002	1.756	0.879‐3.508	.111
High	1	1.111E+08	0‐	.999	1.380E+09	0‐	.999
MONO		2.277	1.053‐4.925	.036	1.482	0.657‐3.341	.343
Normal	463	Ref.			Ref.		
High	44						
EO				.042			.006
Normal	210	Ref.			Ref.		
Low	290	0.606	0.406‐0.905	.014	0.525	0.351‐0.784	.002
High	7	1.239	0.222‐6.927	.807	0.408	0.082‐2.027	.273
BASO		0.192	0.044‐0.833	.027	1.558	0.302‐8.027	.596
Normal	497	Ref.			Ref.		
High	10						
HCT		0.841	0.502‐1.409	.512	0.555	0.359‐0.856	.008
Normal	154	Ref.			Ref.		
Low	353						
MCV				.987			.222
Normal	483	Ref.			Ref.		
Low	13	3.780E+09	0‐	.999	3.925E+09	0‐	.999
High	11	1.143	0.233‐5.609	.869	10.494	0.736‐149.530	.083
MCH				.417			.490
Normal	485	Ref.			Ref.		
Low	12	0.000	0‐	.999	0.000	0‐	.999
High	10	3.542	0.544‐23.063	.186	0.300	0.042‐2.160	.232
MCHC				.948			.755
Normal	481	Ref.			Ref.		
Low	23	1.157	0.367‐3.650	.803	0.823	0.263‐2.580	.739
High	3	1.447	0.046‐45.566	.834	2.876	0.134‐61.970	.500
RDW		1.001	0.528‐1.895	.998	1.353	0.690‐2.654	.378
Normal	436	Ref.			Ref.		
High	71						
MPV		0.373	0.014‐9.990	.557	1.712E+08	0‐	.999
Normal	505	Ref.			Ref.		
High	2						

**Table 5:** Effects of clinical parameters and hemograms on the dysphagia and gastrointestinal reaction in NPC patients (n = 507)

Variables	n	Dysphagia			Gastrointestinal reaction		
		OR	95% CI	*P*‐values	OR	95% CI	*P*‐values
Sex		1.095	0.663‐1.806	.724	0.560	0.345‐0.909	.019
Male	386	Ref.			Ref.		
Female	121						
Age		1.216	0.721‐2.052	.462	1.762	0.996‐3.117	.052
<60 years	389	Ref.			Ref.		
≥60 years	118						
T				.852			.289
T1	62	Ref.			Ref.		
T2	149	0.857	0.419‐1.751	.672	1.108	0.503‐2.437	.800
T3	151	1.012	0.488‐2.102	.974	1.144	0.509‐2.570	.745
T4	145	0.818	0.395‐1.694	.588	0.685	0.310‐1.516	.351
N				.198			.103
N0	40	Ref.			Ref.		
N1	84	0.968	0.374‐2.509	.947	0.969	0.346‐2.713	.953
N2	305	0.590	0.252‐1.381	.224	0.636	0.258‐1.565	.325
N3	78	0.917	0.343‐2.450	.862	1.454	0.485‐4.359	.504
M		1.738	0.918‐3.288	.089	4.129	1.738‐9.807	.001
M0	436	Ref.			Ref.		
M1	71						
Histology				.663			.859
Keratinizing*	12	Ref.			Ref.		
Non‐Keratinizing#	479	0.908	0.231‐3.560	.890	1.106	0.278‐4.404	.886
Unknown	16	0.550	0.098‐3.089	.497	0.807	0.139‐4.675	.811
SIRI		1.073	0.538‐2.136	.842	2.115	1.137‐3.932	.018
<1.529	367	Ref.			Ref.		
≥1.529	140						
NLR		1.150	0.500‐2.643	.742	0.738	0.297‐1.833	.512
<3.441	377	Ref.			Ref.		
≥3.441	130						
SII		1.321	0.579‐3.014	.509	0.933	0.377‐2.308	.881
<715.739	384	Ref.			Ref.		
≥715.739	123						
PLR		2.626	1.304‐5.289	.007	1.825	0.690‐4.822	.225
<245.496	442	Ref.			Ref.		
≥245.496	65						
WBC				.239			.070
Normal	341	Ref.			Ref.		
Low	146	0.787	0.364‐1.704	.544	1.521	0.739‐3.133	.255
High	20	4.568	0.730‐28.573	.104	6.415	1.039‐39.610	.045
RBC				.643			.876
Normal	324	Ref.			Ref.		
Low	178	1.350	0.722‐2.525	.347	0.837	0.425‐1.649	.607
High	5	1.095E+09	0‐	.999	1.990E+08	0‐	.999
HGB		0.920	0.498‐1.699	.790	0.922	0.476‐1.784	.810
Normal	330	Ref.			Ref.		
Low	177						
PLT				.345			.834
Normal	455	Ref.			Ref.		
Low	15	2.132	0.508‐8.948	.301	1.603	0.344‐7.472	.548
High	37	0.633	0.260‐1.545	.316	0.991	0.364‐2.701	.986
NEU				.045			.046
Normal	370	Ref.			Ref.		
Low	109	1.532	0.703‐3.339	.284	1.009	0.466‐2.185	.981
High	28	0.148	0.029‐0.765	.023	0.139	0.029‐0.659	.013
LYM				.340			.967
Normal	379	Ref.			Ref.		
Low	127	1.699	0.838‐3.445	.142	1.103	0.523‐2.328	.797
High	1	2.848E+09	0‐	.999	0.000	0‐	.999
MONO		1.367	0.605‐3.091	.452	0.494	0.223‐1.094	.082
Normal	463	Ref.			Ref.		
High	44						
EO				.008			0.002
Normal	210	Ref.			Ref.		
Low	290	0.536	0.357‐0.806	.003	0.440	0.275‐0.702	.001
High	7	0.334	0.068‐1.640	.177	0.968	0.094‐9.979	.978
BASO		3.606	0.416‐31.228	.244	0.429	0.094‐1.964	.275
Normal	497	Ref.			Ref.		
High	10						
HCT		0.477	0.303‐0.749	.001	0.526	0.320‐0.866	.012
Normal	154	Ref.			Ref.		
Low	353						
MCV				.380			.722
Normal	483	Ref.			Ref.		
Low	13	3.791E+09	0‐	.999	8.486E+08	0‐	.998
High	11	5.437	0.500‐59.136	.164	2.376	0.290‐19.440	.420
MCH				.959			.984
Normal	485	Ref.			Ref.		
Low	12	0.000	0‐	.999	0.000	0‐	.999
High	10	0.766	0.127‐4.609	.771	0.843	0.129‐5.526	.859
MCHC				.831			.525
Normal	481	Ref.			Ref.		
Low	23	1.310	0.401‐4.284	.655	0.880	0.223‐3.477	.855
High	3	1.889	0.089‐40.170	.684	0.128	0.003‐4.683	.263
RDW		1.713	0.935‐3.139	.082	1.156	0.551‐2.426	.701
Normal	436	Ref.			Ref.		
High	71						
MPV		0.104	0.004‐2.806	.178	2.031E+08	0‐	.999
Normal	505	Ref.			Ref.		
High	2						

**Table 6:** Effects of clinical parameters and hemograms on the innutrition in NPC patients (n = 507)

Variables	n	Innutrition		
		OR	95% CI	*P*‐values
Sex		1.397	0.808‐2.417	.232
Male	386	Ref.		
Female	121			
Age		0.589	0.364‐0.952	.031
<60	389	Ref.		
≥60	118			
T				.522
T1	62	Ref.		
T2	149	1.597	0.756‐3.372	.220
T3	151	1.221	0.581‐2.566	.599
T4	145	1.092	0.517‐2.305	.818
N				.863
N0	40	Ref.		
N1	84	0.704	0.279‐1.776	.457
N2	305	0.877	0.377‐2.037	.760
N3	78	0.867	0.326‐2.311	.776
M		0.723	0.380‐1.375	.323
M0	436	Ref.		
M1	71			
Histology				.832
Keratinizing*	12	Ref.		
Non‐Keratinizing#	479	0.592	0.108‐3.228	.544
Unknown	16	0.607	0.077‐4.775	.635
SIRI		0.869	0.419‐1.803	.707
<1.529	367	Ref.		
≥1.529	140			
NLR		1.744	1.044‐2.915	.034
<3.441	377	Ref.		
≥3.441	130			
SII		1.140	0.482‐2.697	.765
<715.739	384	Ref.		
≥715.739	123			
PLR		0.887	0.365‐2.156	.792
<245.496	442	Ref.		
≥245.496	65			
WBC				.913
Normal	341	Ref.		
Low	146	0.918	0.402‐2.097	.840
High	20	1.508	0.195‐11.680	.694
RBC				.084
Normal	324	Ref.		
Low	178	1.728	0.933‐3.198	.082
High	5	0.134	0.009‐2.060	.149
HGB		0.400	0.219‐0.731	.003
Normal	330	Ref.		
Low	177			
PLT				.284
Normal	455	Ref.		
Low	15	5.506	0.668‐45.413	.113
High	37	0.982	0.393‐2.449	.968
NEU				.551
Normal	370	Ref.		
Low	109	0.886	0.391‐2.006	.771
High	28	0.380	0.061‐2.362	.300
LYM				.700
Normal	379	Ref.		
Low	127	1.380	0.653‐2.918	.399
High	1	2.877E+08	0‐	.999
MONO		1.994	0.797‐4.986	.140
Normal	463	Ref.		
High	44			
EO				.874
Normal	210	Ref.		
Low	290	0.948	0.595‐1.510	.822
High	7	0.631	0.101‐3.944	.622
BASO		0.288	0.081‐1.029	.055
Normal	497	Ref.		
High	10			
HCT		0.798	0.449‐1.420	.443
Normal	154	Ref.		
Low	353			
MCV				.089
Normal	483	Ref.		
Low	13	6.745	0.782‐58.147	.082
High	11	4.279	0.527‐34.729	.174
MCH				.900
Normal	485	Ref.		
Low	12	0.000	0‐	.999
High	10	0.627	0.085‐4.619	.647
MCHC				.998
Normal	481	Ref.		
Low	23	0.963	0.298‐3.113	.949
High	3	9.231E+08	0‐	.999
RDW		1.118	0.560‐2.235	.752
Normal	436	Ref.		
High	71			
MPV		9.915E+07	0‐	.999
Normal	505	Ref.		
High	2			

### 3.4 Clinical characteristics of immune‐inflammation indicators in survival analysis

Finally, a total of 255 patients were enrolled in the study for survival analysis. A total of 202 male and 53 female patients in 255 patients with NPC were included (Table 1). Patients’ median age was 51 years (range 12‐78 years). The association between clinical characteristics and immune‐inflammation indicators, such as SIRI, SII, NLR, neutrophil, monocyte and WBC, was shown in Table 7. Among clinical groups of N stage and histology, there were no significant differences in inflammation indicators. We also examined the association between these immune‐inflammation indicators and other haematological indexes. The results showed that there were associations between these indicators and other haematological indicators, including SIRI, NLR, SII, neutrophil, monocyte, WBC and platelet, while most indicators had no difference in RDW. Inflammation indicators also had a significant difference between low and high group of basophils except NLR. Moreover, there was a significant difference between PLR and combined immune indicators such as SIRI, NLR and SII, while no difference in neutrophil, monocyte and WBC.

**Table 7:** Baseline characteristics for patients with SIRI, NLR, SII, Neutrophil, Monocyte and WBC (n = 255)

Variables	SIRI	NLR	SII	Neutrophil	Monocyte	WBC
	<1.529vs ≥1.529	<3.441 vs ≥3.441	<715.739 vs ≥715.739	<2.722 vs ≥2.722	<0.578 vs ≥0.578	<6.177 vs ≥6.177
	*P*	*P*	*P*	*P*	*P*	*P*
Therapy	.759	.208	.277	.120	.603	.126
Untreated						
Chemotherapy						
Radiotherapy						
Sex	.029	.501	.695	.003	.175	.029
Female						
Male						
Age	.010	.093	.054	.064	.120	.433
<60						
≥60						
T	.262	.129	.042	.711	.941	.656
T1						
T2						
T3						
T4						
N	.323	.557	.819	.886	.633	.490
N0						
N1						
N2						
N3						
M	.006	.080	.043	.212	.034	.972
M0						
M1						
Histology	.681	.440	.317	.155	.316	.799
Keratinizing*						
Non‐Keratinizing#						
Unknown						
SIRI		.000	.000	.000	.000	.000
<1.529						
≥1.529						
NLR	.000		.000	.000	.053	.000
<3.441						
≥3.441						
SII	.000	.000		.000	.063	.000
<715.739						
≥715.739						
NEU	.000	.000	.000		.000	.000
<2.722						
≥2.722						
MONO	.000	.053	.063	.000		.000
<0.578						
≥0.578						
WBC	.000	.000	.000	.000	.000	
<6.177						
≥6.177						
PLT	.004	.036	.000	.000	.000	.000
<267.583						
≥267.583						
BASO	.006	.402	.012	.000	.000	.001
<0.029						
≥0.029						
PLR	.000	.000	.000	.354	.243	.967
<245.496						
≥245.496						
RDW	.028	.146	.135	.810	.737	.166
<14.495						
≥14.495						

### 3.5 Associations of immune‐inflammation indicators with survival

**Figure 7:**
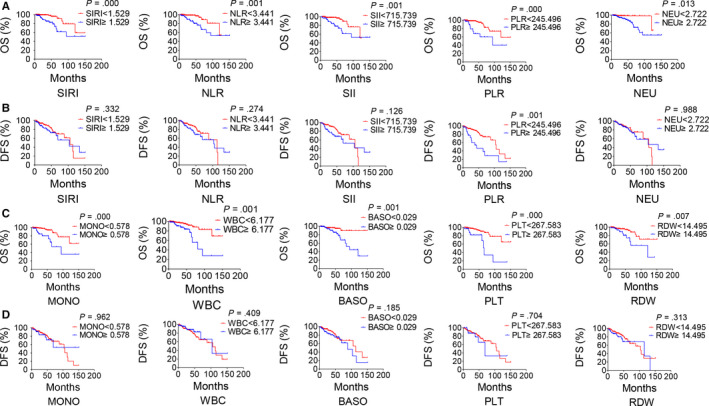
Inflammation indicators predict survival in NPC. Estimated overall survival (OS) (A) and disease‐free survival (DFS) (B) curves for SIRI, NLR, SII and PLR. OS (C) and DFS (D) curves for MONO, WBC, BASO, PLT and RDW. Radiotherapy included radiotherapy alone or chemoradiotherapy

The study took OS and DFS as the primary and secondary outcome, respectively. The median follow‐up time was 33.5 months (range 2.1‐151.2) for OS and 28.4 months (range 1‐151.2) for DFS. Based on the cut‐off values by ROC curve, patients were subdivided into low‐score and high‐score groups of various indicators. Compared with lower scores of haematological indicators, higher scores were associated with significantly worse OS in NPC patients, while it had little effect on DFS except for PLR (Figure 7). By Kaplan‐Meier analysis and the log‐rank test, high‐score SIRI, NLR, SII, neutrophil, monocyte, WBC, platelet, basophil, PLR and RDW were associated with poor OS, while only high‐score PLR was associated with poor DFS (Figure 7). In univariate Cox regression analysis, OS was significantly affected by age, M stage, SIRI, NLR, SII, neutrophil, monocyte, WBC, platelet, basophil, PLR and RDW (Table 8), and DFS was affected by M stage and PLR (Table 9), while the histopathological classification had no effect on OS or DFS. In multivariate Cox regression analysis, for OS, age (*P* = 0.002; HR = 5.061; 95%CI: 1.832‐13.983), M stage (*P* = 0.023; HR = 3.848; 95% CI: 1.204‐12.302), PLR (*P* = 0.035; HR = 3.480; 95%CI: 1.090‐11.117), WBC (*P* = 0.006; HR = 3.500; 95%CI: 1.422‐8.617) and RDW (*P* = 0.008; HR = 3.489; 95%CI: 1.380‐8.818) were independent prognostic risk factors (Table 8). And for DFS, M stage (*P* = .003; HR = 2.862; 95%CI: 1.419‐5.773) and PLR (*P* = 0.017; HR = 2.250; 95%CI: 1.153‐4.394) were independent prognostic risk factors (Table 9).

**Table 8:** Univariate and multivariate Cox proportional hazards regression analysis for OS

Variables	Univariate			Multivariate		
	HR	95% CI	*P*‐values	HR	95% CI	*P*‐values
Therapy			.054			
Untreated	Ref.					
Chemotherapy	0.277	0.017‐4.515	.367			
Radiotherapy	0.086	0.010‐0.718	.023			
Sex	1.218	0.478‐3.103	.679			
Female	Ref.					
Male						
Age	3.091	1.359‐7.033	.007	5.061	1.832‐13.983	.002
<60	Ref.			Ref.		
≥60						
T			.089			
T1	Ref.					
T2	1.087	0.113‐10.492	.942			
T3	1.639	0.191‐14.069	.652			
T4	3.920	0.512‐29.990	.188			
N			.395			
N0	Ref.					
N1	1.681	0.174‐16.210	.653			
N2	1.108	0.144‐8.548	.922			
N3	2.453	0.300‐20.074	.403			
M	4.345	1.837‐10.279	.001	3.848	1.204‐12.302	.023
M0	Ref.			Ref.		
M1						
Histology			.983			
Keratinizing*	Ref.					
Non‐Keratinizing#	6.277E+04	0‐2.123E+275	.972			
Unknown	7.574E+04	0‐2.570E+275	.972			
SIRI	4.355	1.789‐10.600	.001	0.785	0.145‐4.250	.779
<1.529	Ref.			Ref.		
≥1.529						
NLR	4.005	1.633‐9.820	.002	2.354	0.507‐10.935	.275
<3.441	Ref.			Ref.		
≥3.441						
SII	3.717	1.595‐8.658	.002	0.571	0.085‐3.858	.566
<715.739	Ref.			Ref.		
≥715.739						
NEU	5.170	1.210‐22.094	.027	5.821	0.881‐38.448	.067
<2.722	Ref.			Ref.		
≥2.722						
MONO	4.464	1.961‐10.158	.000	1.238	0.338‐4.532	.747
<0.578	Ref.			Ref.		
≥0.578						
WBC	3.864	1.697‐8.801	.001	3.500	1.422‐8.617	.006
<6.177	Ref.			Ref.		
≥6.177						
PLT	4.448	1.881‐10.519	.001	1.354	0.385‐4.760	.637
<267.583	Ref.			Ref.		
≥267.583						
BASO	4.060	1.599‐10.309	.003	1.533	0.511‐4.597	.446
<0.029	Ref.			Ref.		
≥0.029						
PLR	4.123	1.767‐9.617	.001	3.480	1.090‐11.117	.035
<245.496	Ref.			Ref.		
≥245.496						
RDW	2.946	1.290‐6.729	.010	3.489	1.380‐8.818	.008
<14.495	Ref.			Ref.		
≥14.495						

**Table 9:** Univariate and multivariate Cox proportional hazards regression analysis for DFS

Variables	Univariate			Multivariate		
	HR	95% CI	*P*‐values	HR	95% CI	*P*‐values
Therapy			.757			
Untreated	Ref.					
Chemotherapy	1.008	0.000‐4.37E+07	.999			
Radiotherapy	21.961	0.000‐5.20E+07	.680			
Sex	1.345	0.694‐2.604	.380			
Female	Ref.					
Male						
Age	1.080	0.548‐2.130	.824			
<60	Ref.					
≥60						
T			.247			
T1	Ref.					
T2	0.810	0.214‐3.064	.756			
T3	1.442	0.412‐5.043	.567			
T4	1.837	0.541‐6.238	.330			
N			.664			
N0	Ref.					
N1	0.704	0.157‐3.148	.646			
N2	0.895	0.271‐2.960	.856			
N3	1.329	0.363‐4.872	.668			
M	3.672	1.886‐7.149	.000	2.862	1.419‐5.773	.003
M0	Ref.			Ref.		
M1						
Histology			.771			
Keratinizing*	Ref.					
Non‐Keratinizing#	2.318E+04	0‐5.518E+110	.936			
Unknown	3.620E+04	0‐1.516E‐102	.933			
PLR	2.948	1.557‐5.581	.001	2.250	1.153‐4.394	.017
<245.496	Ref.			Ref.		
≥245.496						

## 4 DISCUSSION

In the current study, we found that SIRI, SII, NLR, PLR, neutrophil, monocyte and RDW score were valuable for the prediction of both diagnosis and prognosis of NPC.

Compared with patients with a low score, patients who had a high SIRI score had a shorter OS, as well as SII, NLR, PLR, neutrophil, monocyte, RDW and basophil. Chen et al^20^ also reported the efficacy of SIRI in evaluating the prognosis of NPC, which was consistent with our study. In the univariate Cox regression analysis of our research, inflammation indicators, including SIRI, SII, NLR, PLR, neutrophil, monocyte, RDW and basophils, had a significant correlation with OS, while PLR, WBC, RDW, M stage and age were independent prognostic factors in multivariate Cox regression analysis. The risks of death in patients who attributed to the high‐score groups of the PLR, WBC, RDW, M stage and age were 3.48, 3.5, 3.489, 3.848 and 5.061 times higher than those in the low‐score group of the PLR, WBC, RDW, M stage and age, respectively. Besides, M stage and PLR were also the independent prognostic risk factors for DFS, and the risks of death in the high‐score group of the M stage and PLR were 2.862 and 2.25 times higher than those in the low‐score group of them.

Chronic inflammation plays a vital role in the initiation and development of cancer, which makes individuals susceptible to various types of cancer.^21^ Inflammation was associated with cancer,^22^ such as inflammatory bowel disease with colon cancer, helicobacter pylori infection with gastric cancer and prostatitis with prostate cancer. It has also been reported that patients with chronic rhinosinusitis (CRS) or allergic rhinitis (AR) have increased risk of NPC.^23^ In our study, we compared inflammation indicators of NPC patients with chronic rhinitis patients; then, we conducted a prognostic analysis of haematological indicators for diagnosis of NPC. We found a significant difference between the NPC and rhinitis for immune‐inflammation indicators, such as SIRI, NLR, SII, PLR, neutrophil and monocytes. And PLR was the best predictor of diagnosis of NPC.

Cancers can convert the peripheral matrix to promote progression. The changes involve recruitment of fibroblasts, migration of immune cells and formation of vascular networks. Tumour microenvironment (TME) comprises various cells and extracellular components. Excessive proliferation of cancer cells can stimulate the production of cytokines and chemokines, which attract immune cells to the TME and induce local immune inflammation.^21^ Diem et al reported that NLR and PLR in the tumour microenvironment were associated with prognosis of lung cancer.^24^ In addition, the circulating monocytes that play a major role in innate immunity may reflect the level of tumour‐associated macrophages (TAMs), while TAMs can directly stimulate the growth, migration and metastasis of cancer cells.^25^ Also, the platelet can promote tumour growth and metastasis owing to affecting cancer cells and other cells in the TME.^26^ The different cell types in the TME communicate with each other to support cancer development; for example, SIRI and SII, the combination of NLR and monocyte and platelet, were associated with the prognosis of cancer patients.^19^, ^27^ Neutrophils can promote angiogenesis by pro‐inflammatory cytokines, matrix metalloprotease 9 (MMP9) and VEGF, and can promote tumoral motility, migration and invasion.^28^ Contrary to the pro‐tumour function of neutrophils, monocytes and platelets in malignant carcinomas, lymphocytes play an important role in antitumor immune response.^29^

Most researches have suggested that the neutrophil, monocyte and platelet are pro‐tumour indicators, while lymphocyte regarded as an antitumour indicator. We combine the two or three immunology indicators as prognostic factors, such as SIRI, SII, NLR and PLR, which can enhance the predictive value of the diagnosis and prognosis of tumours. The combined inflammation indicators, low cost and reliable, can be used to supply the current evaluation system of TNM staging system to help evaluate the individualized therapy and prognosis of these patients.

Moreover, RDW is also a potential marker in tumour progression. Mechanically, iron metabolism in red blood cells is affected by inflammatory factors, which induces the release of lots of immature red blood cells from the bone marrow in advance, and inflammatory factors also increase ineffective haematopoiesis in the bone marrow, which together induced a change in the RDW.^30^ Wang et al reported that RDW and body mass index (COR‐BMI) might serve as an inflammation‐ and nutrition‐based indicator of prognosis in NPC.^31^ Consistently, our results showed that RDW might help to predict the diagnosis and prognosis of NPC. The association between basophil and NPC has not been reported so far. In our study, NPC patients with high‐score basophils had poor OS, which testified that basophil might participate in predicting the prognosis of NPC.

Besides, the NPC incidence of males is higher than that of females, and 50‐ to 60‐year‐olds are typical peaks. The ageing of the immune system may result in detrimental consequences on the response against cancers; then, the inflammatory status can promote immune suppression and cancer growth.^32^ In our study, the incidence of NPC in males was three times higher than in females, and the incidence of patients who were under 60 years was three times higher than in those older than 60 years. And the risks of death of patients in the period of older than 60 years were 5.061 times higher than those in lower age.

Radiotherapy can affect the health‐related quality of life (QOL) in patients with NPC, such as dysphagia.^33^ To guarantee the QOL of NPC patients, we investigated the influencing factors for side effects of treatment. We have analysed the influence of clinical parameters and haemograms on side effects in NPC patients based on the reference range of haemogram. The therapies induced most side effects, such as the arrest of bone marrow, radiation stomatitis and dermatitis. Sex, age and M stage have effects on these side effects. Besides, we find that inflammation indicators have significance on various side effects, including the NLR, monocyte, lymphocyte, platelet, eosinophil, basophils, PLR and SIRI.

The summary of the inadequacy of our study is as follows. Most patients with NPC fail to follow‐up, and patients almost diagnosed with non‐keratinizing carcinoma, only 2.4% NPC patients diagnosed with keratinizing squamous cell carcinoma, which may explain why most immunological indicators were not statistically significant in histopathological groups and histology had no effect on side effects and survival in our study. Besides, the items of EB virus load and correlated antibody were regarded as regular tests for patients with NPC in August 2017 in our hospital, while this retrospective study performed in 2014. The correlation between immunological indicators and EBV is not analysed.

In conclusion, the inflammation indicators, such as SIRI, SII, NLR, PLR, neutrophil, monocyte and RDW, can be used to predict the diagnosis and prognosis of NPC. Furthermore, many indicators are closely related to side effects and survival. Because the biological diversity of the tumour has not been taken into account, the current TNM staging system that most common parameters used in therapeutic decision and assessing the curative effect in patients with NPC leads to heterogeneous curative effects in patients with identical TNM staging. The inflammation indicators can replenish the current TNM staging system to help evaluate treatment decision and prognosis. It deserves us to focus on these blood indicators associated with tumour‐related inflammation.

## CONFLICT OF INTEREST

The authors declare that they have no conflict of interest.

## AUTHOR CONTRIBUTIONS

YP conceived and designed the manuscript. XZ, GL and YP acquired, analysed and interpreted the data and wrote and reviewed the manuscript. YL supervised the study.

## Supporting information

Table S1

## Data Availability

The data that support the findings of this study are available on request from the corresponding author. The data are not publicly available due to privacy or ethical restrictions.
