# Supplementary material for: Development and validation of immune inflammation–based index for predicting the clinical outcome in patients with nasopharyngeal carcinoma
Source: J Cell Mol Med. 2020 Jun 30;24(15):8326–49. doi: 10.1111/jcmm.15097 (PMC7412424; doi:10.1111/jcmm.15097)
Supplement: Supplementary file 1 — Table S1 [file JCMM-24-8326-s001.docx]

Table S1 Therapeutic regimens of NPC patients

| Therapeutic regimen | patients (n=559) |
| --- | --- |
| Therapy (on admission ) |  |
| Untreatment | 87 (15.6%) |
| Chemotherapy alone | 41(7.3%) |
| Chem-radiotherapy | 406 (72.6%) |
| Radiotherapy alone | 25 (4.5%) |
| Therapy (out of hospital) |  |
| Untreatment | 4 (0.7%) |
| Chemotherapy alone | 46 (8.2%) |
| Chem-radiotherapy | 468 (83.7%) |
| Radiotherapy alone | 41 (7.4%) |
